# Modelling Neglected Tropical Diseases diagnostics: the sensitivity of skin snips for *Onchocerca volvulus* in near elimination and surveillance settings

**DOI:** 10.1186/s13071-016-1605-3

**Published:** 2016-06-14

**Authors:** Christian Bottomley, Valerie Isham, Sarai Vivas-Martínez, Annette C. Kuesel, Simon K. Attah, Nicholas O. Opoku, Sara Lustigman, Martin Walker, Maria-Gloria Basáñez

**Affiliations:** MRC Tropical Epidemiology Group, London School of Hygiene and Tropical Medicine, Keppel Street, London, WC1E 7HT UK; Department of Statistical Science, University College London, Gower Street, London, WC1E 6BT UK; Cátedra de Salud Pública. Facultad de Medicina (Escuela Luis Razetti), Universidad Central de Venezuela, Caracas, Venezuela; UNICEF/UNDP/World Bank/WHO, Special Programme for Research and Training in Tropical Diseases, World Health Organization, Geneva, Switzerland; University of Health and Allied Sciences Research Centre (UHASRC) Hohoe, Volta Region, Ghana; Department of Microbiology, University of Ghana Medical School, Accra, Ghana; Laboratory of Molecular Parasitology, Lindsley F. Kimball Research Institute, New York Blood Center, 310 E 67th St, New York, NY 10065 USA; London Centre for Neglected Tropical Disease Research, Department of Infectious Disease Epidemiology, School of Public Health, Faculty of Medicine (St Mary’s campus), Norfolk Place, London, W2 1PG UK

**Keywords:** Onchocerciasis, Sensitivity, Skin snips, Ivermectin, Elimination, Surveillance, Prevalence, Diagnostics

## Abstract

**Background:**

The African Programme for Onchocerciasis Control has proposed provisional thresholds for the prevalence of microfilariae in humans and of L3 larvae in blackflies, below which mass drug administration (MDA) with ivermectin can be stopped and surveillance started. Skin snips are currently the gold standard test for detecting patent *Onchocerca volvulus* infection, and the World Health Organization recommends their use to monitor progress of treatment programmes (but not to verify elimination). However, if they are used (in transition and in parallel to Ov-16 serology), sampling protocols should be designed to demonstrate that programmatic goals have been reached. The sensitivity of skin snips is key to the design of such protocols.

**Methods:**

We develop a mathematical model for the number of microfilariae in a skin snip and parameterise it using data from Guatemala, Venezuela, Ghana and Cameroon collected before the start of ivermectin treatment programmes. We use the model to estimate sensitivity as a function of time since last treatment, number of snips taken, microfilarial aggregation and female worm fertility after exposure to 10 annual rounds of ivermectin treatment.

**Results:**

The sensitivity of the skin snip method increases with time after treatment, with most of the increase occurring between 0 and 5 years. One year after the last treatment, the sensitivity of two skin snips taken from an individual infected with a single fertile female worm is 31 % if there is no permanent effect of multiple ivermectin treatments on fertility; 18 % if there is a 7 % reduction per treatment, and 0.6 % if there is a 35 % reduction. At 5 years, the corresponding sensitivities are 76 %, 62 % and 4.7 %. The sensitivity improves significantly if 4 skin snips are taken: in the absence of a permanent effect of ivermectin, the sensitivity of 4 skin snips is 53 % 1 year and 94 % 5 years after the last treatment.

**Conclusions:**

Our model supports the timelines proposed by APOC for post-MDA follow-up and surveillance surveys every 3–5 years. Two skin snips from the iliac region have reasonable sensitivity to detect residual infection, but the sensitivity can be significantly improved by taking 4 snips. The costs and benefits of using four versus two snips should be evaluated.

**Electronic supplementary material:**

The online version of this article (doi:10.1186/s13071-016-1605-3) contains supplementary material, which is available to authorized users.

## Background

Onchocerciasis intervention programmes in Africa changed their focus from reducing skin and ocular morbidity associated with the infection - and therefore controlling the disease as a public health problem - to eliminating the infection reservoir [1, 2], after both mathematical models of transmission [3] and field studies [4–6] showed that local elimination of *Onchocerca volvulus* can be achieved through community-directed treatment with ivermectin (CDTI). The current goal is to eliminate onchocerciasis in selected African countries by 2020 [7] and in 80 % of endemic countries by 2025 [8].

In response to the focus on elimination, the African Programme for Onchocerciasis Control (APOC) has proposed provisional parasitological and entomological thresholds for stopping CDTI [9]. Specifically, APOC has suggested that CDTI can be stopped if, 11–12 months after the last ivermectin treatment round, microfilarial prevalence (i.e. the proportion of people with *O. volvulus* microfilariae in the skin based on two iliac crest skin snips) is lower than 5 % in all surveyed villages and lower than 1 % in 90 % of the villages, and the prevalence of infective vectors (simuliid flies) is less than 0.5 infective flies per 1000 flies. Once treatment has stopped, APOC recommends that a follow-up survey is conducted after 3 years, and then regular surveillance every 3–5 years. Although still advocating parasitological testing using skin snips for monitoring progress towards elimination, the World Health Organization (WHO) has recently released guidelines [10], proposing that PCR-based xenomonitoring in blackfly samples and seropositivity to the Ov-16 antigen in children aged < 10 years should be used to demonstrate interruption of transmission for the purpose of stopping treatment. Certification of elimination will therefore probably be based on entomological and serological evaluations. Nonetheless in African countries approaching elimination, a modification of the “skin snip method” that was employed by the Onchocerciasis Control Programme in West Africa (OCP) [11, 12] remains the cornerstone of epidemiological evaluations of infection prevalence. For example, between 2008 and 2015, 58 APOC-led CDTI projects underwent epidemiological evaluations using the skin snip protocol [6, 13]. Indeed, a WHO/APOC document on alternative treatment strategies for accelerating progress towards elimination in Africa [14], released in December 2015, reiterates the practice of skin snipping to monitor progress towards elimination and confirm when treatment can be safely stopped without excessive risk of infection recrudescence (so-called phase 1a and phase 1b evaluations, respectively).

Although other diagnostics for patent infection (i.e. presence of macrofilariae capable of producing microfilariae) are available - notably the 'diethylcarbamazine (DEC) patch test' and the detection of *O. volvulus* DNA in skin snips via polymerase chain reaction (PCR) [15–20] - and despite limitations [19, 21, 22], the skin snip method is still considered the gold standard for diagnosing patent infection and measuring the number of *O. volvulus* microfilariae in the skin.

Typically two skin snips are taken with a 2-mm Holth-type corneoscleral punch and incubated in a suitable medium (usually saline), ideally for 24 hours [23]. In Africa, both snips are taken from the iliac crest (hip). In Meso- and South American foci, a shoulder-scapular-snip has sometimes replaced one of the iliac crest snips in view of the greater density of microfilariae in the upper torso in areas with upper body biting vectors [24, 25]. After incubation, the microfilariae that have emerged from the snips are counted using an inverted microscope and reported as the arithmetic or geometric mean per snip (mf/ss). When the snips are weighed, the mean number of microfilariae is expressed per mg of skin (mf/mg).

As is the case for all diagnostic tests, the accuracy of the skin snip method is determined by its sensitivity (the probability that an infected individual tests positive) and specificity (the probability that an uninfected individual tests negative). The specificity of the skin snip method is likely to be close to 100 %, unless the microfilariae from other filarial parasites (e.g. *Mansonella streptocerca*, also skin-dwelling, and *M. perstans* and *M. ozzardi*, which are blood-dwelling) are incorrectly identified as *O. volvulus.* The sensitivity, however, is less than 100 % and depends on a number of factors, including the number of snips examined, the number of fertile female worms harboured, the distribution of microfilariae in the skin, the snip incubation medium and duration, the host immune response, and the thoroughness with which the sample is examined under the microscope [20–22, 26–30]. When an individual has received treatment, the sensitivity also depends on the anti-onchocercal drug used and the time since treatment. In the context of evaluating CDTI, the impact of ivermectin treatment on the sensitivity is of particular interest.

In this study we use a mathematical model to predict the sensitivity of the skin snip method in a hypothetical community that has received CDTI. We use our model to explore how many skin snips should be taken to monitor progress towards elimination and to suggest when post-treatment surveillance surveys should be done.

## Methods

### Study areas and parasitological methods for the datasets analysed

We used data collected from four studies. Two were conducted in Latin America - in the central focus of Guatemala in the vicinity of Lake Atitlán [24] and in the Amazonian focus of southern Venezuela [31, 32], and two were conducted in Africa - in the Volta region of Ghana [33] and in the Kumba region of Cameroon [34].

#### Meso-American setting

The Guatemalan data were collected as part of a study to assess the impact of 6-monthly ivermectin treatment on the prevalence, intensity of infection, and transmission in the central Guatemalan onchocerciasis focus [24, 35]. We used data from the pre-treatment, baseline, survey, which was conducted in May 1988. The study included the villages of Los Andes, Los Tarrales, Santa Emilia, El Vesubio and Santa Isabel (for an epidemiological description of this focus prior to mass drug administration with ivermectin see Brandling-Bennett et al. [36]). A map indicating the location of these communities is available in Fig. 1 of Collins et al. [24]. For the analyses presented here, we excluded data from El Vesubio because of its small population size and from Santa Isabel because there was poor compliance with skin snipping in this village.

#### South-American setting

The Venezuelan data were collected during an epidemiological survey of onchocerciasis among Yanomami communities in the Amazonian onchocerciasis focus [30–32]. A map indicating the location of the study communities is provided in Fig. 1 of [31]. We used data from 14 villages that contributed to the Venezuelan study, namely Aweitheri, Cerrito, Hasupiwei, Hokotopiwei, Kumamasi, Mahekoto, Maiyotheri, Maweti, Pashopëka, Purimatheri, Toothothopiwei, Yepropë, Yoreashiana A and Yoreashiana B.

#### African forest setting

The Ghanaian data were collected in a baseline survey for a randomised trial designed to assess the safety of moxidectin as a treatment for *O. volvulus* infection [33]. The study was conducted before CDTI, and participants were recruited from onchocerciasis endemic villages in a forest area within the River Tordzi basin in the Volta Region of Ghana. Ninety percent of participants came from the villages of Honuta-Gbogame, Kpedze-Anoe, Togorme, Aflakpe, Luvudo, Kpoeta-Ashanti and Hoe, the remainder came from 11 other villages in the area. A map indicating the location of the villages is shown in Fig. 3 of [33]. At the time of the study (2006), the area was not included in the CDTI strategy of the National Onchocerciasis Control Programme because overall it was classified as hypoendemic in the Rapid Epidemiological Mapping of Onchocerciasis (REMO) [33]. The few meso- and hyperendemic villages would have been missed by the survey.

The Cameroonian data were collected in a hyperendemic forest area of Kumba in southwest Cameroon before the implementation of CDTI. The study participants had not previously received ivermectin, and were born or had resided for more than 10 years in the villages of Marumba I, Marumba II, Boa Bakundu, Bombanda and Bombele [34].

In the Guatemalan and Venezuelan studies, two skin snips were taken from each participant: one from the left shoulder and the other from the left iliac crest. In Ghana and Cameroon, one skin snip was taken from each calf and iliac crest (i.e. four skin snips in total). In all studies, a 2.0-mm corneoscleral Holth or Walser punch was used to obtain the samples. In Guatemala,Venezuela and Cameroon only the Holth corneoscleral punch was used, while in Ghana both types of punch were used*.* The samples were subsequently incubated for 24 h, or at least 8 h (overnight) in Ghana, in physiological saline solution. Although the incubation time in Ghana was less than in the other studies, according to [23] 97 % of the microfilariae would have emerged after 8 h of incubation. The methods used for counting microfilariae and processing and weighing skin snips are described in [33, 36, 37]. Table 1 summarises demographic and parasitological characteristics of the participants in these studies.

**Table 1:** Demographic and parasitological characteristics of the datasets used to parameterise the model

Country	No. of villages	*n* ^a^	Median (Interquartile range) age (years)	% Female	Location of skin snips	Mean microfilarial load (mf/mg skin)	Mean weight of skin snips (mg)	References
Guatemala	3	1,067	19 (10, 36)	44.2	1 shoulder & 1 hip	shoulder = 26.3 hip = 25.4	shoulder = 1.35 hip = 1.80	[24]
Venezuela	14	613	22 (12, 35)	39.8	1 shoulder & 1 hip	shoulder = 14.2 hip = 31.9^c^	shoulder = 1.32 hip = 1.73	[30–32]
Ghana	18	172^b^	17–60^b^	18.2–31.1	2 hip & 2 calf	hip = 27.4 calf = 16.6^c^	hip = 2.15 calf = 2.14	[33]
Cameroon	5	2,528	17 (10, 35)	49.1	2 hip & 2 calf or 2 calf	hip = 8.0 calf = 9.5	Not weighed^d^	[34]

^a^ *n*, number of individuals whose skin snip data were used in the model

^b^Participants were allocated to four treatment groups. The mean age in the four groups ranged from 32.1 to 38.3 years. The table indicates the minimum and maximum age across all treatment groups

^c^ *P*-value for comparison of microfilarial load between the two body sites: *P* < 0.001 Venezuela; *P* < 0.001 Ghana

^d^Skin snips were not weighed in Cameroon, so it was assumed that a skin snip taken from the hip weighed 2.2 mg, and that one taken from the calf weighed 2.1 mg based on the data from Ghana

### Modelling the sensitivity of skin snips

To estimate the sensitivity of the skin snip method we must model both the density and distribution of microfilariae in the skin. If microfilariae are randomly distributed in the skin, then we can use the Poisson distribution to calculate the sensitivity directly from the density of microfilariae. For example, if a single 2-mg skin snip is taken from an individual who has on average 0.5 microfilariae per mg of skin, then the expected number of microfilariae in the sample (*m*) is 0.5 × 2 = 1. And, from the Poisson assumption, it follows that the probability the skin snip contains no microfilariae is exp(-*m*) = exp(-1) = 0.37 and the probability the sample contains one or more microfilariae, i.e. the sensitivity, is 1–0.37 = 0.63.

However, microfilariae are unlikely to be randomly distributed in the skin (e.g. the density may depend on the distribution of fertile worms in the body). If the distribution is non-random because microfilariae occur in “clumps”, then this will affect the sensitivity of skin snips because the chance that a snip contains no microfilariae is increased. To allow for this, we can use the negative binomial model rather than the Poisson to model the distribution of microfilariae in the skin. This distribution is specified by an additional parameter, *k*, which measures the degree of aggregation, i.e. the extent of the clumping. The distribution is approximately Poisson when *k* is large, but becomes more aggregated (overdispersed) as *k* gets closer to 0. Continuing the example of *m* = 1 above, if *k* = 0.4 then from the negative binomial distribution the probability the skin snip contains no microfilariae is ${\left(1+\frac{m}{k}\right)}^{-k}=0.61$ and the sensitivity is therefore 1–0.61 = 0.39.

Below we first estimate the extent to which microfilariae are aggregated in the skin (i.e. we estimate the *k* parameter) using existing data that were collected prior to widespread ivermectin treatment. We then use this estimate, together with a model of microfilarial density after treatment, to estimate the sensitivity of the skin snip methodology after treatment. The model of microfilarial density is parameterised using estimates for pre-treatment microfilarial production, resumption of microfilarial production after treatment and microfilarial mortality that have been reported elsewhere [38–42].

### A mathematical model of the number of microfilariae per skin snip before treatment

We model the number of microfilariae, *x*_*ij*_, in the *i* th skin snip from site *j* (shoulder, iliac crest or calf) as an immigration-death process. According to this model, microfilariae arrive in the skin at a body site-specific rate *ε*_j_^*^ per milligram of skin per fertile female worm and die at per capita rate *μ*_*m*_ [43, 44]. In individuals who have not received anti-microfilarial treatment, the number of microfilariae in a skin snip follows a Poisson distribution with mean *m*_ij_^*^ given by *m*_ij_^*^ = *d*_*ij*_*wε*_j_^*^/*μ*_*m*_, where *d*_*ij*_ is the weight of the skin snip and *w* the number of fertile female worms harboured by the host.

The rate at which microfilariae arrive at the skin is likely to vary according to the location of the skin snip [45]. This is because adult worms (macrofilariae) are aggregated into onchocercal nodules (onchocermata), which are themselves not evenly distributed within the body and vary in their distribution among individuals [38, 46]. We represent this additional variability by a scaling factor that has a gamma distribution with mean one and shape parameter *k*_*m*_, which we apply to *m*_ij_^*^. It follows that the marginal, pre-treatment distribution of microfilarial load per skin snip follows a negative binomial distribution with mean *m*_ij_^*^ and dispersion parameter *k*_*m*_. For skin snips taken from a host harbouring *w* fertile female worms, the probability of sampling *x*_*ij*_ microfilariae in the *i* th skin snip biopsy from site *j* is therefore

1 $$ \mathrm{p}\left({x}_{ij}\Big|w\right)\kern0.5em =\kern0.5em \frac{\varGamma \left({k}_m\kern0.5em +\kern0.5em {x}_{ij}\right)}{x_{ij}\kern0.1em !\varGamma \left({k}_m\right)}{\left(1\kern0.5em +\kern0.5em \frac{m_{ij}^{*}}{k_m}\right)}^{-\kern0.5em \left({k}_m\kern0.5em +\kern0.5em {x}_{ij}\right)}{\left(\frac{m_{ij}^{*}}{k_m}\right)}^{x_{ij}}\kern0.4em , $$

where Γ(*z*) is the gamma function.

### Fitting the model to pre-treatment data

We used the method of maximum likelihood to estimate the parameters of the model from data on pre-treatment microfilarial loads. The model was fitted separately to data from each country.

The probability of the microfilarial loads observed in *a single* host can be obtained by averaging over the (unobserved) distribution of fertile female worms (*w*), assuming it follows a negative binomial distribution within each village [40], with mean *θ*_*w*_ and dispersion parameter *k*_*w*_:

2 $$ {\displaystyle \sum_w{\displaystyle \prod_{ij}\;\mathrm{p}\left({x}_{ij}\left|w\right.\right)\ \mathrm{p}(w)}}. $$

And the probability of the data as a whole- i.e. the likelihood - is the product of these probabilities. We estimated the model parameters by maximising the logarithm of this likelihood using the maxLik package in R [47]. Specifically, we used the likelihood to estimate, *θ*_*w*_ and *k*_*w*_ in each village, the ratio of body site-specific microfilarial production (*α* = *ε*_1_^*^/*ε*_2_^*^), and the aggregation of microfilariae in the skin (*k*_*m*_). Since the latter depends on the number of fertile female worms [46, 48], we allowed *k*_*m*_ to depend on *w* and obtained estimates for 1–10, 11–30 and > 30 fertile female worms.

The data could not be used to estimate the rate of microfilarial mortality or the rate at which microfilariae arrive in the skin. We therefore fixed *μ*_*m*_ = 0.8 per year [38, 39] and ε* = (*ε*_1_^*^ + *ε*_2_^*^) /2 = 1.154 per year [41]. The latter represents the average of the two body site-specific rates in an untreated individual (shoulder and iliac crest in Guatemala and Venezuela, and calf and iliac crest in Ghana and Cameroon), and the value was obtained by scaling the average annual rate of microfilarial production per female worm per mg of skin presented in [39] by the reciprocal of the proportion of females that are fertile [40].

### Sensitivity of multiple skin snips as a test for infection with *O. volvulus*

To predict the sensitivity of the skin snip method when used in a population that has been treated with ivermectin, we consider a person infected with a *single* fertile female worm and model the effect of treatment with ivermectin on the density of microfilariae in the person’s skin. Together with the estimate of microfilarial aggregation in the skin, we use the microfilarial density to predict the probability that a skin snip contains one or more microfilariae. This model of sensitivity implicitly assumes that all microfilariae that emerge from the skin snip are detected, although in practice the sensitivity will also depend on how thoroughly the sample is examined under the microscope.

#### Microfilaricidal and embryostatic effects of ivermectin

Treatment with ivermectin has two effects on the number of microfilariae found in the skin. First, microfilariae are paralysed and move to deeper body organs where they are destroyed (the so-called microfilaricidal effect). This results in the almost total clearance of microfilariae from the skin within the first one or two months of treatment. Second, adult female worms temporarily stop producing live microfilariae, as newly produced microfilariae are blocked inside the uterus (the so-called embryostatic effect). This leads to the suppression of microfilaridermia for several months [40], although the rate at which microfilariae disappear from and reappear in the skin differs somewhat between individuals [33].

The embryostatic effect is well documented, but it is unclear whether microfilarial production eventually returns to its original level or whether the fertility of some adult female worms is permanently impaired after each treatment [41, 43, 49–53]. Plaisier et al. [49] test two hypotheses for the effect of ivermectin on microfilarial production: the first assumes that treatment has only a transient effect on the microfilarial production; the second assumes that in addition to the transient effect, ivermectin also causes a permanent reduction in microfilarial production.

We allow for both possibilities by assuming that after exposure to *n* rounds of ivermectin a fertile female worm resumes production and release of microfilariae at rate (1 − *ζ*)^*n*^*ε*_j_^*^, after a period of time that is exponentially distributed with rate *ρ* = 0.29 year^−1^ [40, 42], where *ζ* is the per dose reduction in fertility caused by the treatment. According to this model, there is a permanent as well as a transient effect when *ζ* > 0, but only a transient effect when *ζ* = 0. Based on these assumptions, the expected rate of microfilarial production at time *t* after the last dose of ivermectin is

3 $$ {\varepsilon}_j(t)={\left(1-\zeta \right)}^n{\varepsilon}_j^{*}\kern0.5em \left[1- \exp \left(-\rho\;t\right)\right]. $$

And the expected number of microfilariae in a skin snip skin taken from an individual infected with a *single* female worm *t* years after the last treatment is

4 $$ {m}_{ij}(t)=\frac{d_{ij}{\left(1-\zeta \right)}^n{\varepsilon}_j^{*}}{\mu_m\left(\rho -{\mu}_m\right)\;}\left\{\;\rho\;\left[1- \exp \left(-{\mu}_mt\right)\right]-{\mu}_m\left[1- \exp \left(-\rho\;t\right)\right]\right\}\;. $$

If we assume that the microfilarial load in a skin snip follows a negative binomial with mean *m*_*ij*_(*t*), and denote the event that any of the skin snips are positive by *x*^+^(*t*), then the sensitivity of a test for onchocerciasis that combines multiple skin snips taken *t* years after the last treatment is

5 $$ \mathrm{p}\left({x}^{+}(t)\Big|w\kern0.5em =\kern0.5em 1\right)=\kern0.5em 1\kern0.5em -\kern0.5em \underset{i}{\Pi}\underset{j}{\Pi}\mathrm{p}\left({x}_{ij}(t)\kern0.5em =\kern0.5em 0\Big|\kern0.5em w\kern0.5em =\kern0.5em 1\right)\kern0.5em =\kern0.5em 1\kern0.5em -\kern0.5em \underset{i}{\Pi}\underset{j}{\Pi}\kern0.5em {\left(1\kern0.5em +\kern0.5em \frac{m_{ij}(t)}{k_m}\right)}^{-{k}_m}. $$

When infection with more than one fertile female worm is rare - mathematically this is the case when *θ*_*w*_/*k*_*w*_ < < 1 - the proportion of the population that tests positive is approximately the product of the sensitivity and the proportion infected, since

6 $$ \mathrm{p}\left({x}^{+}\right)\kern0.5em =\kern0.5em \underset{w}{\varSigma}\kern0.5em p\left({x}^{+}\Big|w\right)p(w)\kern0.5em \approx \kern0.5em p\left({x}^{+}\Big|w\kern0.5em =\kern0.5em 1\right)p\left(w\kern0.5em =\kern0.5em 1\right)\kern0.5em \approx \kern0.5em p\left({x}^{+}\Big|w\kern0.5em =\kern0.5em 1\right)p\left(w\kern0.5em >\kern0.5em 0\right). $$

We use eqn [5] to investigate three scenarios for the impact of treatment with ivermectin on sensitivity: 1) treatment does not have a cumulative effect on microfilarial production (*ζ* = 0) [43]; 2) treatment reduces microfilarial production by 7 % per dose (*ζ* = 0.07) [54]; and 3) treatment reduces microfilarial production by 35 % per dose (*ζ* = 0.35) [42, 49, 55]. We further assume that in a hypothetical community that has received prolonged CDTI, a fertile female worm has been exposed to *n* = 10 annual treatment rounds. This number of treatments was chosen because the reproductive life span of female worms is 9–11 years [56], so under annual CDTI it is unlikely that a worm is exposed to a greater number of treatments. (Under a biannual treatment strategy, a worm can be exposed to twice the number of treatments, but we do not explore this scenario.) The model parameters are defined in Table 2.

**Table 2:** Notation, definition and values of model parameters

Symbol	Definition	Value and units	References
*x*_*ij*_	Number of microfilariae in the *i*th skin snip biopsy from body site *j* (shoulder, hip or calf)	Data from each participant	[24, 30–34]
*d*_*ij*_	Weight of the *i* th skin snip biopsy from body site *j* (shoulder, hip or calf)	See Table 1, in mg	[24, 30–33]
ε*	Rate of production of microfilariae per (mated) fertile worm per mg of skin	1.1538 year^-1^	[41]
*m*_ij_^*^	Mean number of microfilariae per skin snip at baseline	For the mean number of mf/mg for each site and setting see Table 1	
*k*_*m*_	Aggregation (overdispersion) parameter of skin microfilariae	Estimated by fitting the model, see Table 3	
*μ*_*m*_	Per capita rate of microfilarial mortality	0.8 year^-1^	[38, 39]
*w*	Number of adult (mated) fertile female worms harboured by the host	Unobserved, assumed to be linearly related to microfilarial load	[39]
*θ*_*W*_	Mean number of adult (mated) fertile female worms per host	Unobserved, estimated by fitting the model, see Table 3	
*k*_*W*_	Aggregation (overdispersion) parameter of adult female worms	Estimated by fitting the model, see Table 3	
*α* = *ε*_1_^*^/*ε*_2_^*^	Ratio of the rate of microfilarial arrival in a shoulder or calf skin snip to the rate in a snip from the iliac crest	Estimated by fitting the model, see Table 3	
*ρ*	Rate of resumption of microfilarial production	0.29 year^-1^	[40, 42]
*t*	Time since last ivermectin treatment	1, 3, 5 years	
*ζ*	The per dose reduction in female worm fertility caused by ivermectin treatment	0	[43]
		0.07	[54]
		0.35	[55]
*n*	No. of (annual) ivermectin rounds to which a surviving (mated) female worm has been exposed	10	
*x* ^+^	The event that any of the skin snips taken from a host are positive		

## Results

### Model parameter estimates

We fitted the model to skin snip data collected from 4,380 individuals before community-wide distribution of ivermectin; 46 % were female and the median age was 18 years (interquartile range, IQR: 10–35 years, Table 1). The model-estimated mean burden of fertile female worms and aggregation varied considerably between villages, particularly in Venezuela, where the mean burden was 0.4/host in Yepropë and 50 in Aweitheri, and *k*_*w*_ varied from 0.02 (most aggregated) in Yepropë to 0.59 (least aggregated) in Pashopëka (Table 3). In contrast, the aggregation of microfilariae in the skin was similar across the countries and there was a consistent pattern of increased aggregation with decreasing worm burden (i.e. microfilariae were less evenly distributed in the skin at low worm burdens). Across the 4 studies, the average aggregation parameter for skin microfilariae, *k*_*m*_, was 0.42 for 1–10 female worms, 0.76 for 11–30 worms and 1.82 for > 30 female worms.

**Table 3:** Parameter estimates obtained by fitting a model for the number of microfilariae in a skin snip to data collected in four countries (notation as in Table 2)

		Guatemala	Venezuela	Ghana	Cameroon
Aggregation of microfilariae as a function of (unobserved) female worm burden^a^	*k*_*m*_				
1–10 adult female worms		0.35 (0.27, 0.46)	0.48 (0.32, 0.73)	0.54 (0.41, 0.70)	0.29 (0.26, 0.32)
11–30 adult female worms		0.60 (0.47, 0.76)	0.50 (0.31, 0.80)	1.25 (1.01, 1.55)	0.69 (0.56, 0.85)
>30 adult female worms		1.42 (1.13, 1.78)	1.35 (1.00, 1.82)	2.75 (1.74, 4.34)	1.77 (1.45, 2.17)
Mean burden of fertile female worms (range across villages)	*θ*_*W*_	15–19	0.4–50	15^b^	5–16
Aggregation of female worm burden (range across villages)	*k*_*W*_	0.38–0.52	0.02–0.59	2.27^b^	0.31–0.50
Ratio of microfilarial arrival rate in the two body sites sampled per setting (relative to iliac crest)^a^	*α*	1.08 (0.95, 1.22) (shoulder/hip)	0.34 (0.28, 0.43) (shoulder/hip)	0.57 (0.49, 0.66) (calf/hip)	1.0 (0.81, 1.21) (calf/hip)

^a^Estimates presented with 95 % Wald confidence interval

^b^Too few data per village for village-specific estimates

### Sensitivity of 1, 2, 4 and 6 skin snips *t* years after the last ivermectin treatment

We used the average aggregation in microfilarial load (*k*_*m*_ = 0.42), estimated from the pre-treatment data for infections of 1–10 fertile female worms, and eqns [4] and [5] to predict the sensitivity of 1, 2, 4 and 6 skin snips (assuming a weight of 2 mg per snip) taken from an individual infected with a single fertile female worm *t* years after the last ivermectin treatment. Apart from *k*_*m*_, the parameter values used in eqn [4] (i.e. ε*= 1.154 per worm per mg of skin per year; *μ*_*m*_= 0.8 per year, and *ρ* = 0.29 per year) were estimated in previous studies (Table 2).

**Fig. 1:**
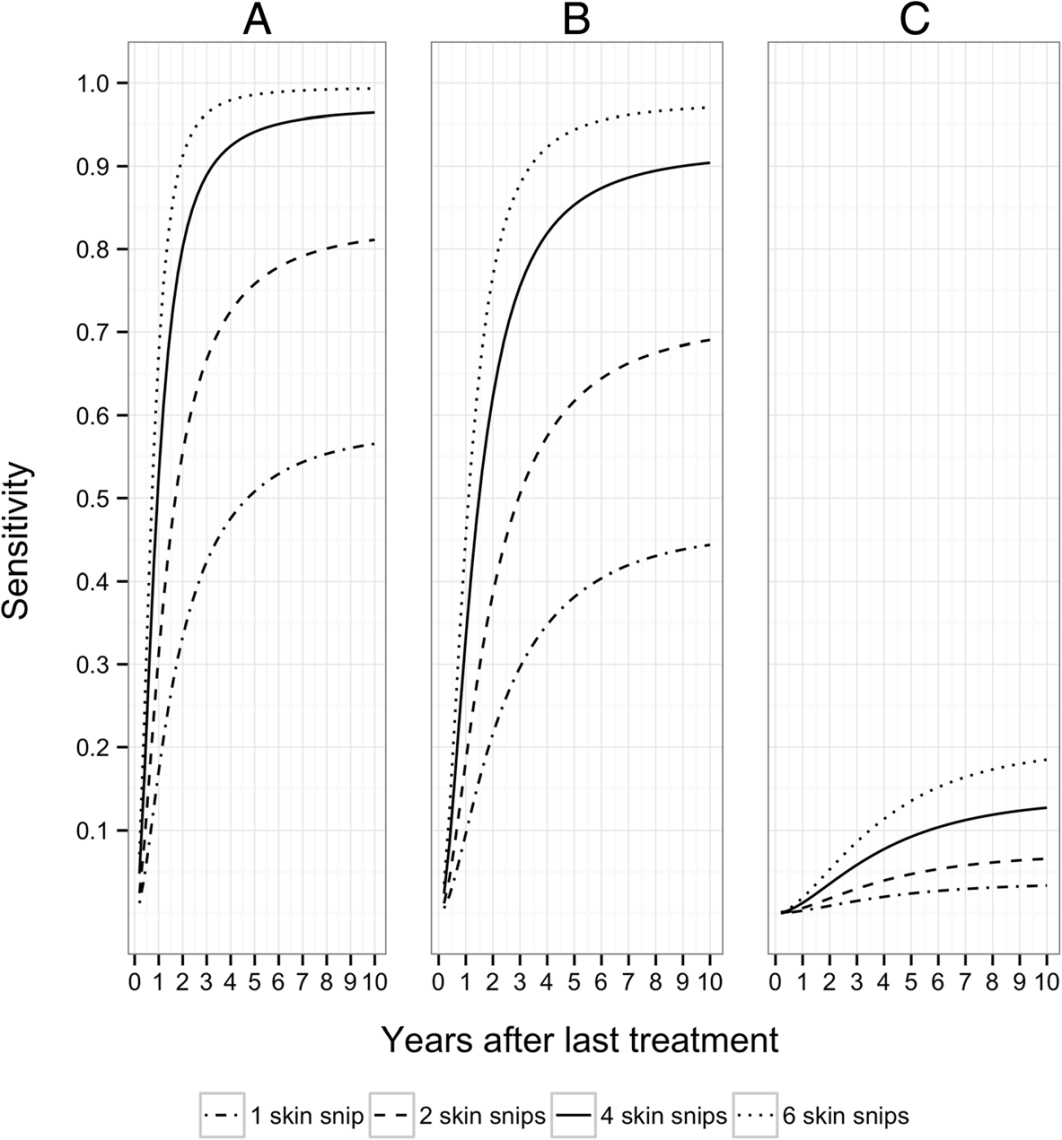
Sensitivity of 1, 2, 4 and 6 skin snips (assuming a weight of 2 mg per snip) taken from an individual infected with a single, fertile female worm. Three scenarios are explored for the effect of ivermectin on microfilarial production: (**a**) microfilarial production by adult female worms is independent of the number of previous exposures to ivermectin (i.e. *ζ*=0 [43]); (**b**) each round of treatment reduces microfilarial production by 7 % (*ζ*=0.07 [54]); (**c**) each treatment round reduces production by 35 % (*ζ*=0.35 [55]). It is assumed that the worms have been exposed to 10 rounds of (annual) ivermectin treatment. Other parameter values are: microfilarial aggregation in the skin, *k*_*m*_ = 0.42 (mean of the country-specific estimates for 1–10 adult female worms), pre-treatment microfilarial production per fertile female worm per mg of skin per year, ε* = 1.154 (estimate from [41]), microfilarial mortality per year *μ*_*m*_ = 0.8 (estimate from [38, 39]), and resumption of microfilarial production per year *ρ* = 0.29 (estimate from [40, 42]). The dot-dash lines correspond to 1 snip; the dashed lines to 2 snips; the solid lines to 4 snips and the dotted lines to 6 snips

We found that the sensitivity of one or more skin snips increases with time after treatment, with most of the increase occurring between 0 and 5 years (Fig. 1). This is because the rapid and almost complete clearance of microfilariae soon after treatment is followed by the resumption of production by adult female worms, and subsequent release of microfilariae, which increases rapidly up to 5 years.

**Fig. 2:**
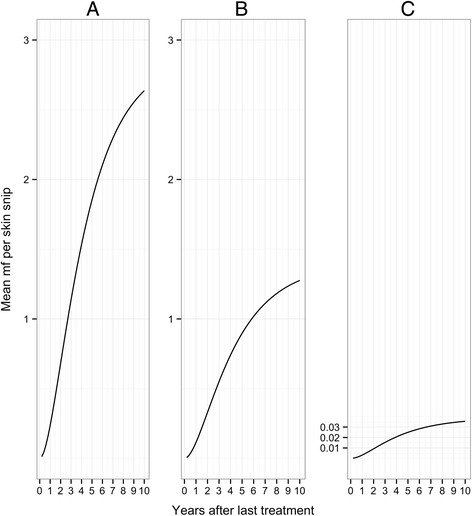
Mean microfilarial load in a single skin snip taken from an individual infected with a single, fertile female worm. Panels (**a**) to (**c**) and parameter values are as defined in Fig. 1

**Fig. 3:**
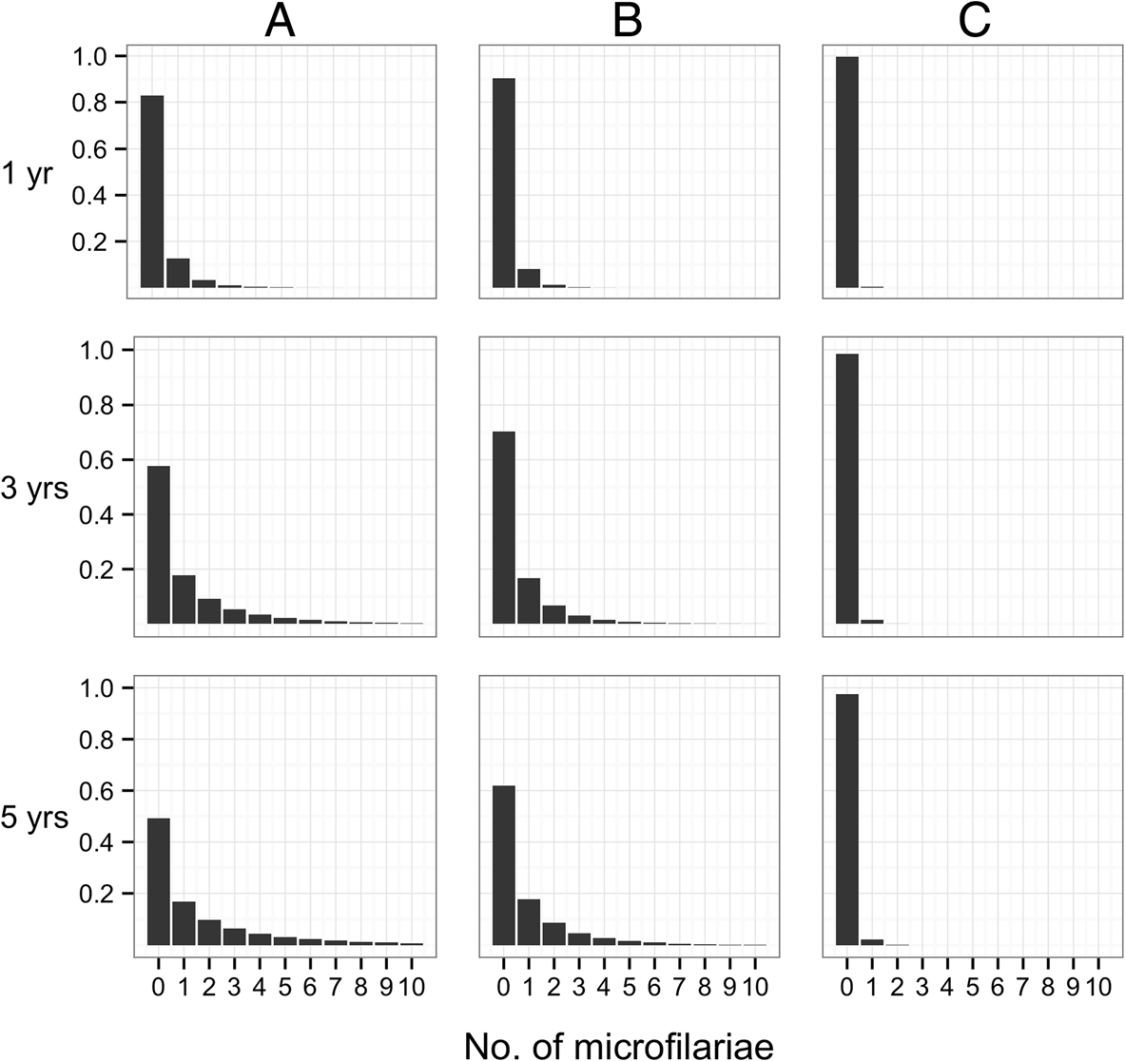
Frequency distribution of microfilarial load among skin snips taken from an individual infected with a single, fertile adult female worm. The vertical, *y*-axis, represents the proportion of snips with the number of microfilariae represented on the horizontal, *x*-axis. Rows correspond to times after the last treatment with ivermectin (upper row = 1 year; middle row = 3 years; bottom row = 5 years). Panels (**a**) to (**c**) and parameter values are as defined in Fig. 1

The change in sensitivity thus mirrors the production of microfilariae and consequently depends on the assumptions made about the impact of ivermectin on the fertility of adult female worms. Figures 2 and 3 show how the mean number of microfilariae per skin snip and the frequency distribution of microfilariae among snips change with time.

One year after treatment, the sensitivity of two skin snips taken from an individual infected with a single fertile female worm is 31 %, assuming no permanent effect of multiple ivermectin treatments on fertility (*ζ* = 0); 18 % assuming 7 % reduction per treatment (*ζ* = 0.07), and 0.6 % assuming a 35 % reduction per treatment (*ζ* = 0.35). At 5 years, the estimates are 76 %, 62 % and 4.7 %, respectively. The sensitivity improves significantly if 4 skin snips are taken. For example, if there is no permanent effect of ivermectin on adult female worm reproduction, the sensitivity of 4 skin snips is 53 % 1 year after the last treatment and 94 % at 5 years. Estimates of the sensitivity of 1, 2, 4 and 6 skin snips at 1, 3, and 5 years are presented in Table 4.

**Table 4:** Sensitivity of 1, 2, 4 and 6 skin snips taken 1, 3 and 5 years after the last ivermectin treatment

		Sensitivity (%)^a^		
		Time after last treatment		
		1 year	3 years	5 years
0 % reduction in microfilarial production per treatment				
	Number of skin snips			
	1	17	42	51
	2	31	67	76
	4	53	89	94
	6	67	96	99
7 % reduction in microfilarial production per treatment				
	Number of skin snips			
	1	10	30	38
	2	18	51	62
	4	33	76	85
	6	45	88	94
35 % reduction in microfilarial production per treatment				
	Number of skin snips			
	1	0.3	1.5	2.4
	2	0.6	3.0	4.7
	4	1.3	5.8	9.2
	6	1.9	8.6	13.5

^a^Estimates of sensitivity are based on *k*_*m*_ = 0.42 (mean of the country-specific estimates for 1–10 adult female worms).

In Venezuela and Ghana, the density of microfilariae was greater in skin snips taken from the iliac crest than in snips taken from the second skin site, namely, the scapular in Venezuela and the calf in Ghana (Table 1 and Table 3). In Additional file 1: Figure S1 and S2, we therefore present body site-specific estimates of sensitivity for these countries, which show that sensitivity is highest when skin snips are taken from the iliac crest.

Although our estimates of microfilarial aggregation in the different studies were similar, they may not be generalizable to other settings. In Additional file 1: Figure S3 we therefore show how the sensitivity varies for *k*_*m*_ in the range 0.01 to 1. It can be seen that skin snips become less sensitive as the aggregation increases (*k*_*m*_ decreases).

### Microfilarial prevalence

We can use eqn [6] and estimates of skin snip sensitivity to improve estimates of the microfilarial prevalence of *O. volvulus* when infection is rare. Table 5 presents prevalence estimates when the 2-snip skin snip method indicates a 0.25 %, 0.5 % or 1 % microfilarial prevalence at 1 year, 3 years and 5 years after the last ivermectin treatment.

**Table 5:** Predicted estimates of prevalence (%) of patent infection for a given (2-snip) sensitivity and observed prevalence of microfilariae

Per ivermectin dose reduction in microfilarial production	1 year after last treatment			3 years after last treatment			5 years after last treatment		
	Sensitivity^a^	Prevalence (%)		Sensitivity^a^	Prevalence (%)		Sensitivity^a^	Prevalence (%)	
		Obs^b^	Pred		Obs^b^	Pred		Obs^b^	Pred
0 % reduction per treatment									
	31 %	0.25	0.8	67 %	0.25	0.4	76 %	0.25	0.3
		0.5	1.6		0.5	0.7		0.5	0.7
		1.0	3.2		1.0	1.5		1.0	1.3
7 % reduction per treatment									
	18 %	0.25	1.4	51 %	0.25	0.5	62 %	0.25	0.4
		0.5	2.8		0.5	1.0		0.5	0.8
		1.0	5.6		1.0	2.0		1.0	1.6
35 % reduction per treatment									
	0.6 %	0.25	na^c^	3.0 %	0.25	8.3	4.7 %	0.25	5.3
		0.5	na^c^		0.5	na^c^		0.5	na^c^
		1.0	na^c^		1.0	na^c^		1.0	na^c^

^a^Sensitivity for 2 skin snips based on *k*_*m*_ = 0.42 (mean of the country-specific estimates for 1–10 adult female worms)

^b^Observed prevalence based on 2 skin snips

^c^ *na* not applicable, we do not present predicted prevalence when greater than 10 % since eqn [6] is only valid when infection is rare

## Discussion

In this paper, we used a mathematical model of the density and distribution of *O. volvulus* microfilariae in the skin to predict the sensitivity of the skin snip method for identifying patent *O. volvulus* infection in communities that have received CDTI and where the infection is close to elimination.

We fitted the model to pre-treatment skin snip data from two Latin American [24, 31] and two African [33, 34] studies, and obtained estimates of the mean female worm burden per person, the aggregation in worm burden, the aggregation of microfilariae in skin snips, and the ratio of microfilarial density in the two different regions of the body from which the skin snips were taken. Ideally we would have included in our analysis data collected in the savannah (the African data analysed in this paper were collected in forest foci [33, 34]), but the available microfilarial count data (e.g. from northern Cameroon, analysed in [39]) were aggregated across skin snips, and the snips were not weighed. One of the differences between forest and savannah parasites is that their microfilariae are located at different depths in the dermis-microfilariae have a more superficial distribution in the forest than in the savannah [57] - which might affect skin snip sensitivity.

Our estimates of mean *fertile female* worm burden per host for Guatemala (15–19) and Ghana (15) are in line with estimates of *female* worm burden based on nodulectomy data from a forest focus in Liberia (26 female worms per person, including live and dead worms), and a savannah focus in Burkina Faso (24 female worms) [46], since approximately 60 % of female worms are fertile [40]. They are also consistent with model-derived estimates from the same data (24 female worms in men and 23 in women, in Liberia; 17 female worms in men and 19 in women, in Burkina Faso) [48]. It should be noted that the data analysed did not include observed numbers of female worms (e.g. from data on excision of onchocercomata (nodulectomy) or nodule palpation). Our estimates of fertile female worm burden are indirect estimates that have been derived assuming that there is a linear (density independent) relationship between worm burden and microfilarial load [41, 48, 54, 58].

The estimated aggregation of microfilariae within a skin snip site was consistent across the four studies. However, the skin snip data used to parameterise the model were mainly collected from hyperendemic villages, with the exception of the Venezuela Amazonian focus where endemicities ranged from hypoendemic (Purima, Yepropë), and mesoendemic (Mahekoto, Maweti, Toothothopiwei) to hyperendemic-and we therefore cannot be certain that the estimated relationship between microfilarial aggregation in the skin and worm burden applies where there is a low intensity of infection (e.g. after long-term CDTI). Since we observed that the degree of aggregation increases as worm burden declines, we might expect that in these settings microfilariae are more aggregated and skin snips are less sensitive [41, 54, 55]. In the future, we hope that our methodology can be used to estimate the degree of microfilarial aggregation from microfilarial count data obtained from hypoendemic settings as they are incorporated into treatment programmes [59].

We estimated that the density of microfilariae was higher in the iliac region than in the scapular region in Venezuela, and that it was higher in the iliac region than in the calves in Ghana. This implies that among skin snips of comparable size taken from individuals in Venezuela and Ghana, those taken from the iliac crest are more sensitive than those taken from the other body site. And since our data show that iliac skin snips are also heavier than other skin snips we expect an even greater difference in sensitivity per skin snip [21, 29]. The difference in microfilarial distribution across body sites was not observed in the other two countries (Guatemala and Cameroon), which suggests that the distribution of microfilariae across the body varies with geographical location, and possibly reflects the region most frequently bitten by the prevailing simuliid vectors [60, 61].

Following [21, 29] we explored the sensitivity of 1 to 6 skin snips and confirmed that sensitivity can be greatly improved by taking multiple skin snips. However, communities are increasingly reluctant to participate in skin snipping, so a compromise must be found between what is feasible and the ideal. Currently, two un-weighed iliac snips are taken during APOC surveys, but if four could be taken the sensitivity of the diagnostic would be greatly improved. For example, one and three years after treatment, four skin snips have approximately twice and 1.5 times the sensitivity of two snips. Although we found that taking 6 skin snips further increases sensitivity, the extra gain is probably not sufficiently large to warrant the greater inconvenience to the patient.

As intervention programmes progress from control to elimination, APOC has recommended that countries consider alternative treatment strategies [14]. In areas where onchocerciasis is co-endemic with loiasis (African eye worm), ivermectin CDTI needs to be implemented with special precautions because of the risk of severe adverse events among co-infected individuals [62–64]. Currently, a strategy of 'test-and-not treat', identifying those individuals with a high microfilarial load (e.g. ≥ 30,000 microfilariae of *L. loa* per ml of blood), and excluding them from ivermectin treatment is being trialled [65]. However, withdrawing treatment from this group may prohibit onchocerciasis elimination (Stolk et al., unpublished results), particularly if the proportion of non-compliers (those who consistently refuse to take ivermectin) is already high [41, 54, 55, 58, 66]. In these circumstances, test-and-treat protocols are required so that co-infected individuals can be offered alternative therapeutics. One such therapy is doxycycline (or other tretracycline antibiotic), which has macrofilaricidal efficacy against *O. volvulus* but does not affect *L. loa* [67–69]. In areas where loiasis is not co-endemic with onchocerciasis, test-and-treat strategies might be cost effective in situations where there has been prolonged CDTI, but where elimination thresholds have not been achieved because compliance has been poor [14].

Ideally test-and-treat strategies and surveillance should be implemented using tests that are less invasive than the skin snip method. The DEC-patch test ([15–18, 20] and see also [70–72]), and Ov-16 serology [73, 74] are two possible alternatives. The DEC patch test consists of applying diethylcarbamazine citrate topically to a 6 cm^2^ area of skin. The killing of microfilariae induced by DEC leads to a diagnostic skin reaction, which has been validated as a surrogate marker of patent infection [70–72]. A patch that uses Nivea lotion and is applied to the iliac crest has been evaluated in central Africa and was adopted by the OCP for surveillance [15–17], and a patch that incorporates transdermal delivery technology [18] was used in the recent surveillance studies in Mali and Senegal [5]. The Ov-16 antibody test is another alternative, but it has the drawback that it detects past as well as current infection. However, it could be used to detect recrudescence by examining people born after the interruption of transmission [75]. For reviews of alternative diagnostic methods, including biomarkers of adult worm infection, we refer the reader to [19, 22, 73, 76].

## Conclusions

An accurate test (or battery of tests) for patent *O. volvulus* infection is required to monitor progress towards elimination, and to implement alternative treatment strategies. The development of such a test should be a priority. However, the skin snip method currently remains the gold standard since it is the only test that is able to accurately quantify the intensity of infection [14, 22]. The recent WHO guidelines for stopping MDA and verifying elimination of human onchocerciasis [10] state that parasitological evaluation by skin snip microscopy and the DEC-patch test can be used to monitor progress during the first (treatment) phase of onchocerciasis elimination programmes and, although the procedure is not advocated to verify elimination, skin snip evaluation by PCR is recommended to differentiate between active infection and past exposure to the parasite in those situations where Ov-16 seropositivity is at least 0.1 %. It is therefore important that enough skin snips are taken per individual to diagnose *O. volvulus* infections reliably, particularly if a test-and-treat strategy is to replace long-term CDTI (e.g. in areas where targeted treatment may be more cost effective than CDTI), or is implemented in areas where onchocerciasis is hypoendemic.

### Abbreviations

APOC, African Programme for Onchocerciasis Control; CDTI, community-directed treatment of ivermectin; DEC, diethylcarbamazine citrate; IRB, institutional review board; MDA, mass drug administration; mf, microfilariae; mg, milligram; OCP, Onchocerciasis Control Programme in West Africa; PCR, polymerase chain reaction; REMO, rapid epidemiological mapping of onchocerciasis; ss, skin snip; WHO, World Health Organization

## Additional file

Additional file 1: Additional figures illustrating setting-specific sensitivity and the influence of microfilarial overdispersion. **Figure S1.** Sensitivity of the skin snip method in the Amazonian focus of southern Venezuela. **Figure S2.** Sensitivity of the skin snip method in the Volta region of Ghana. **Figure S3.** Sensitivity of the skin snip method under different scenarios for the amount of microfilarial aggregation. (PDF 421 kb)
