# Supplementary material for: Modelling Neglected Tropical Diseases diagnostics: the sensitivity of skin snips for Onchocerca volvulus in near elimination and surveillance settings
Source: Parasit Vectors. 2016 Jun 14;9:343. doi: 10.1186/s13071-016-1605-3 (PMC4908809; doi:10.1186/s13071-016-1605-3)
Supplement: Additional file 1: — Additional figures illustrating setting-specific sensitivity and the influence of microfilarial overdispersion. Figure S1. Sensitivity of the skin snip method in the Amazonian focus of southern Venezuela. Figure S2. Sensitivity of the skin snip method in the Volta region of Ghana. Figure S3. Sensitivity of the skin snip method under different scenarios for the amount of microfilarial aggregation. (PDF 421 kb) [file 13071_2016_1605_MOESM1_ESM.pdf]

Additional File 1

Modelling Neglected Tropical Diseases diagnostics: the sensitivity of skin snips for *Onchocerca volvulus* in near elimination and surveillance settings

Christian Bottomley[1,*], Valerie Isham[2], Sarai Vivas-Martínez[3], Annette C. Kuesel[4], Simon K. Attah[6], Nicholas O. Opoku[5], Sara Lustigman[7], Martin Walker[8,§], Maria-Gloria Basáñez[8,§]

[1] MRC Tropical Epidemiology Group, London School of Hygiene and Tropical Medicine, Keppel Street, London WC1E 7HT, UK

[2] Department of Statistical Science, University College London, Gower Street, London WC1E 6BT, UK

[3] Cátedra de Salud Pública. Facultad de Medicina (Escuela Luis Razetti), Universidad Central de Venezuela, Caracas, Venezuela

[4] UNICEF/UNDP/World Bank/ WHO, Special Programme for Research and Training in Tropical Diseases, World Health Organization, Geneva, Switzerland.

[5] University of Health and Allied Sciences Research Centre (UHASRC) Hohoe, Volta Region, Ghana.

[6] Department of Microbiology, University of Ghana Medical School, Accra, Ghana

[7] Laboratory of Molecular Parasitology, Lindsley F. Kimball Research Institute, New York Blood Center, 310 E 67th St, New York NY10065, USA

[8] London Centre for Neglected Tropical Disease Research, Department of Infectious Disease Epidemiology, School of Public Health, Faculty of Medicine (St Mary's campus), Norfolk Place, London W2 1PG, UK

[*]Corresponding Author: Christian Bottomley; Email: christian.bottomely@lshtm.ac.uk
[§]Joint last authors

Additional figures illustrating setting-specific sensitivity and the influence of microfilarial overdispersion

Figure S1. Sensitivity of the skin snip method in the Amazonian focus of southern Venezuela

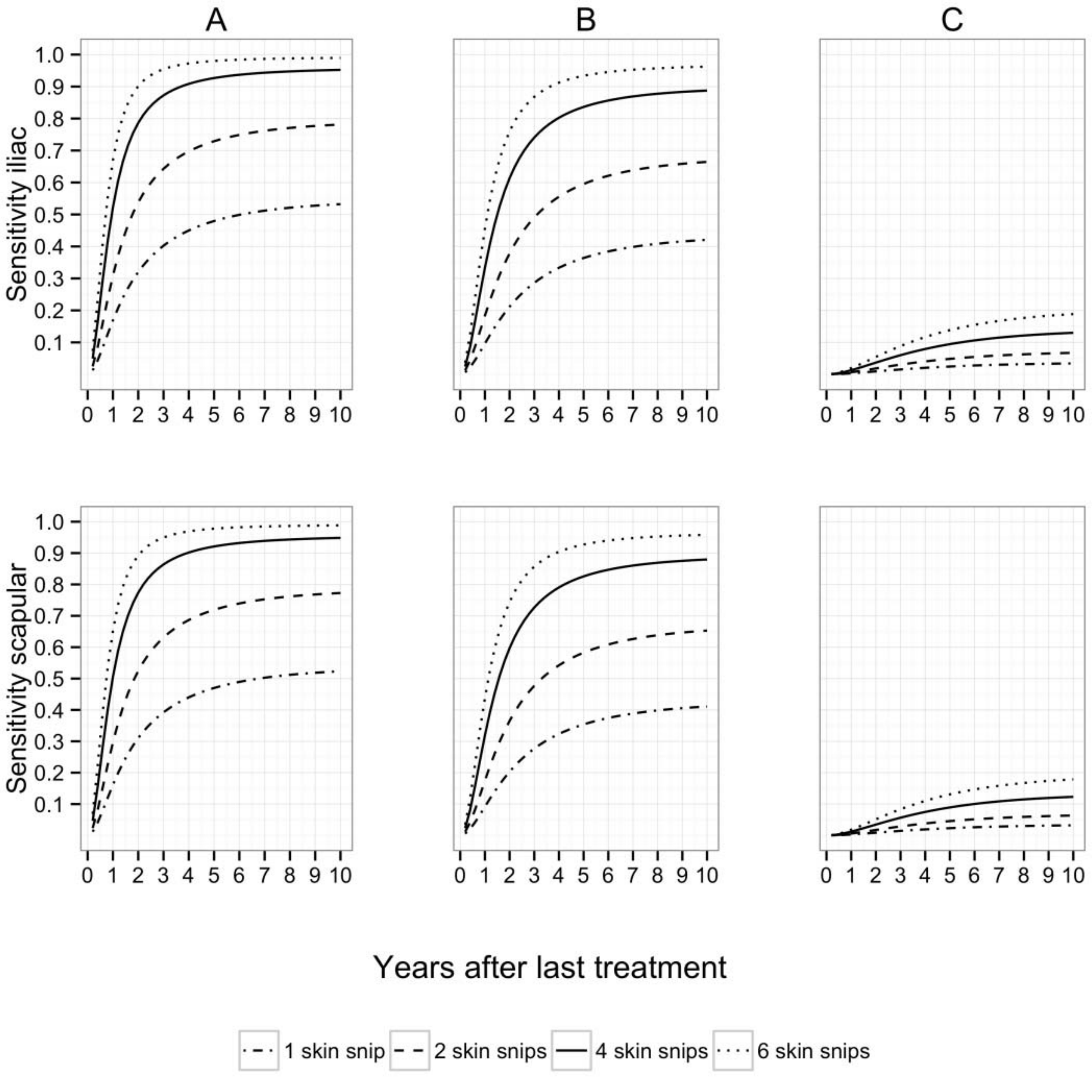

The sensitivity of skin snips taken from the iliac crest (top row) is compared with the sensitivity of snips taken from the scapular region (bottom row). Three scenarios are explored for the effect of ivermectin on microfilarial production: (A) microfilarial production by adult female worms is independent of the number of previous exposures to ivermectin (i.e., ζ =0 [1]); (B) each round of treatment reduces microfilarial production by 7% (ζ =0.07 [2]); (C) each treatment round reduces

production by 35% (ζ =0.35 [3]). It is assumed that the worms have been exposed to 10 rounds of (annual) ivermectin treatment. Other parameter values are: microfilarial aggregation in the skin, k_m = 0.48 (Venezuela-specific estimate for 1–10 adult female worms), microfilarial mortality per year μ_m = 0.8 (estimate from [4, 5]), resumption of microfilarial production per year ρ = 0.29 (estimate from [6, 7]), pre-treatment microfilarial production per fertile female worm per mg of skin in the iliac crest per year, $\varepsilon^{iliac} = 1.722$, in the scapula $\varepsilon^{scapula} = 0.586$ (estimated using $\alpha = \varepsilon^{scapula} / \varepsilon^{iliac} = 0.34$ from Table 3 in main text, and $\left(\varepsilon^{scapula} + \varepsilon^{iliac}\right)/2 = \varepsilon^{*} = 1.154$ [8]). The dot-dash lines correspond to 1 snip; the dashed lines to 2 snips; the solid lines to 4 snips and the dotted lines to 6 snips.

Figure S2. Sensitivity of the skin snip method in the Volta region of Ghana

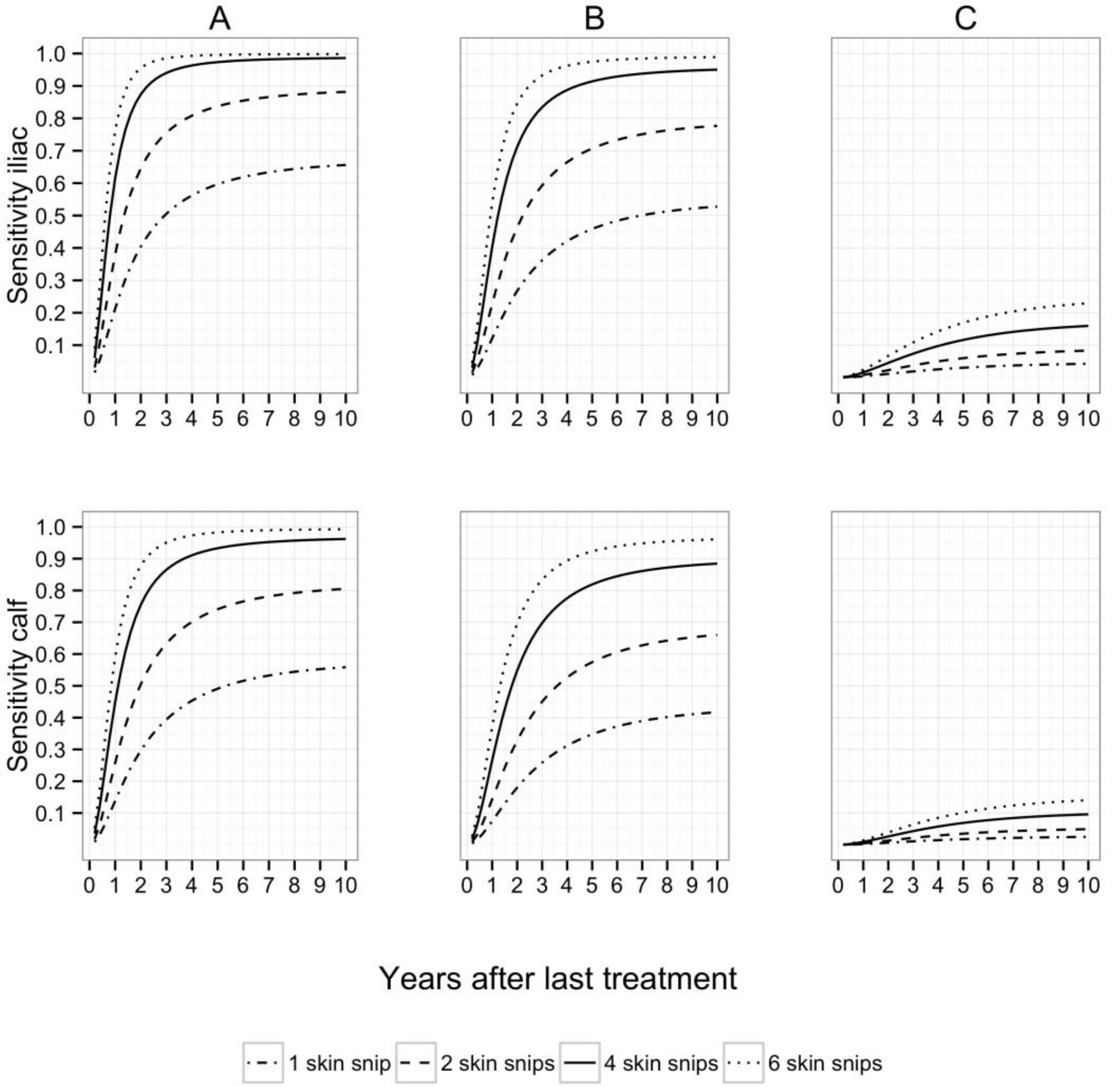

The sensitivity of skin snips taken from the iliac crest (top row) is compared with the sensitivity of snips taken from the calf (bottom row). Panels A to C are as defined in Figure S1. Other parameter values are: microfilarial aggregation in the skin, k_m = 0.54 (Ghana-specific estimate for 1–10 adult female worms), pre-treatment microfilarial production per fertile female worm per mg of skin in the iliac crest per year, $\varepsilon^{iliac} = 1.47$, in the calf $\varepsilon^{calf} = 0.838$ (estimated using $\alpha = \varepsilon^{calf} / \varepsilon^{iliac} = 0.57$ from Table 3 in main text, and $\left(\varepsilon^{calf} + \varepsilon^{iliac}\right)/2 = \varepsilon^{*} = 1.154$ [8]). The dot-dash lines correspond to 1 snip; the dashed lines to 2 snips; the solid lines to 4 snips and the dotted lines to 6 snips.

Figure S3. Sensitivity of the skin snip method under different scenarios for the amount of microfilarial aggregation

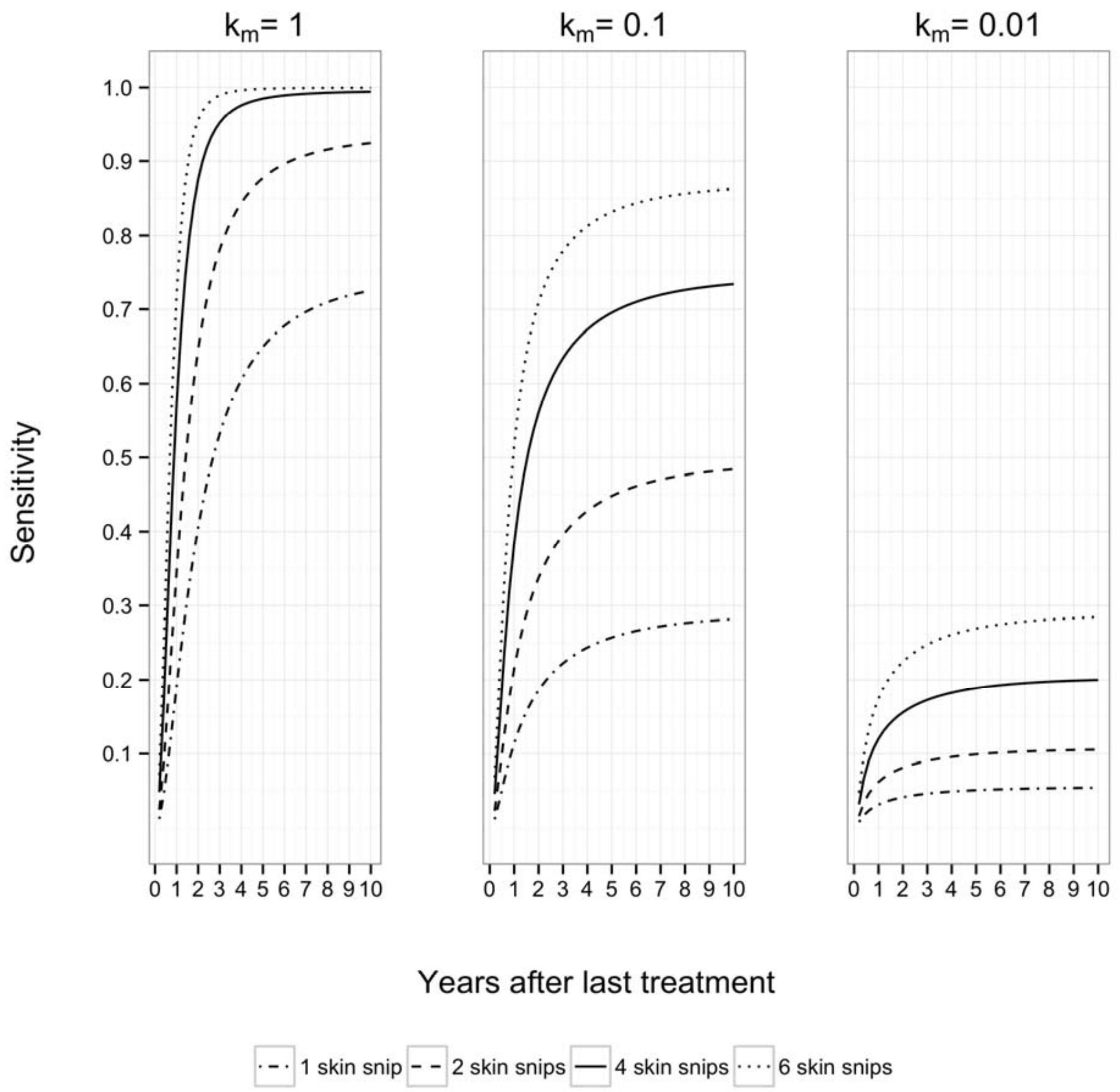

The sensitivity of the skin snip method as a function of time (number of years) after the last ivermectin treatment is compared for k_m = 1, k_m = 0.1 and k_m = 0.01 to explore the influence of an increasing degree of microfilarial overdispersion. Other parameter values are: pre-treatment microfilarial production per fertile female worm per mg of skin per year, $\varepsilon^* = 1.154$ (estimate from [8]), microfilarial mortality per year μ_m = 0.8 (estimate from [4, 5]), and resumption of microfilarial production per year ρ = 0.29 (estimate from [6, 7]). The dot-dash lines correspond to 1 snip; the dashed lines to 2 snips; the solid lines to 4 snips and the dotted lines to 6 snips.

References

1. Bottomley C, Isham V, Collins RC, Basáñez MG. Rates of microfilarial production by *Onchocerca volvulus* are not cumulatively reduced by multiple ivermectin treatments. Parasitology. 2008; 135(13):1571–1581.

2. Turner HC, Walker M, Churcher TS, Basáñez MG. Modelling the impact of ivermectin on River Blindness and its burden of morbidity and mortality in African Savannah: EpiOncho projections. Parasit Vectors. 2014; 7:241.

3. Plaisier AP, Alley ES, Boatin BA, Van Oortmarssen GJ, Remme H, De Vlas SJ, Bonneux L, Habbema JDF. Irreversible effects of ivermectin on adult parasites in onchocerciasis patients in the Onchocerciasis Control Programme in West Africa. J Infect Dis. 1995; 172(1):204–210.

4. Duke BOL. The population dynamics of *Onchocerca volvulus* in the human host. Trop Med Parasitol. 1993; 44(2):61–68.

5. Basáñez MG, Boussinesq M. Population biology of human onchocerciasis. Philos Trans R Soc Lond B Biol Sci. 1999; 354(1384):809–826.

6. Basáñez MG, Pion SDS, Boakes E, Filipe JAN, Churcher TS, Boussinesq M. Effect of single-dose ivermectin on *Onchocerca volvulus*: a systematic review and meta-analysis. Lancet Infect Dis. 2008; 8(5):310–322.

7. Coffeng LE, Stolk WA, Hoerauf A, Habbema D, Bakker R, Hopkins AD, de Vlas SJ. Elimination of African onchocerciasis: modeling the impact of increasing the frequency of ivermectin mass treatment. PLoS One. 2014; 9(12):e115886.

8. Turner HC, Churcher TS, Walker M, Osei-Atweneboana MY, Prichard RK, Basáñez MG. Uncertainty surrounding projections of the long-term impact of ivermectin treatment on human onchocerciasis. PLoS Negl Trop Dis. 2013; 7(4):e2169.
